# Screening and Validation of Internal Reference Genes for Quantitative Real-Time PCR Analysis of Leaf Color Mutants in *Dendrobium officinale*

**DOI:** 10.3390/genes14051112

**Published:** 2023-05-19

**Authors:** Hua Cao, Han Li, Lin Lu, Yulu Ji, Lulin Ma, Shenchong Li

**Affiliations:** 1 Flower Research Institute of Yunnan Academy of Agricultural Sciences, Kunming 650205, China; 2 Fujian Forestry Science and Technology Experimental Center, Zhangzhou 363600, China; 3 National Engineering Technology Research Center for Ornamental Horticulture, No. 2238 Beijing Road, Kunming 650204, China

**Keywords:** *Dendrobium officinale*, leaf color, qRT-PCR, reference gene, leaf color mutant

## Abstract

Leaf color mutants (LCMs) are important resources for studying diverse metabolic processes such as chloroplast biogenesis and differentiation, pigments’ biosynthesis and accumulation, and photosynthesis. However, in *Dendrobium officinale*, LCMs are yet to be fully studied and exploited due to the unavailability of reliable RGs (reference genes) for qRT-PCR (quantitative real-time reverse transcription PCR) normalization. Hence, this study took advantage of previously released transcriptome data to select and evaluate the suitability of ten candidate RGs, including Actin (*Actin*), polyubiquitin (*UBQ*), glyceraldehyde-3-phosphate dehydrogenase (*GAPDH*), elongation factor 1-α (*EF1α*), β-tubulin (*β-TUB*), α-tubulin (*α-TUB*), 60S ribosomal protein L13-1 (*RPL13AD*), aquaporin PIP1-2 (*PIP1-2*), Intima protein (*ALB3*) and Cyclin (*CYCB1-2*) for normalizing leaf color-related genes’ expression levels via qRT-PCR. Stability rankings analysis via common software Best-Keeper, GeNorm, and NormFinder disclosed that all ten genes met the requirements of RGs. Of them, *EF1α* exhibited the highest stability and was selected as the most reliable. The reliability and accuracy of *EF1α* were confirmed through qRT-PCR analysis of fifteen chlorophyll pathway-related genes. The expression patterns of these genes via *EF1α* normalization were consistent with the results by RNA-Seq. Our results offer key genetic resources for the functional characterization of leaf color-related genes and will pave the way for molecular dissection of leaf color mutations in *D. officinale*.

## 1. Introduction

Leaf color mutations are common and easily detectable morphological phenotype variations in higher plants [1,2]. They are usually expressed at the seedling stage and are generally categorized into many types, including greenish-white, albino, light green, greenish-yellow, white emerald, etiolation, striped, yellow-green, purple, dark green, and brown [3,4,5]. Besides, LCMs can also be classified into four types: total chlorophyll deficient type, total chlorophyll increased type, chlorophyll a deficient type, and chlorophyll b deficient type [6]. Genes involved in leaf color often play critical roles in the biosynthesis of photosynthetic pigments and chloroplast development [1,6]. Therefore, LCMs represent essential resources for insight into plant physiology and metabolism as color mutations mostly affect plants’ photosynthetic efficiency, which results in poor growth and development performances and considerable economic losses [1,2]. Consequently, LCMs have become ideal materials for investigating pigments’ metabolism, chloroplast formation and differentiation, photosynthesis, biotic and abiotic stress responses, etc. [1,2]. In rice, studies on LCMs have significantly contributed to the crop improvement [1]. For instance, LCMs and senescence-associated mutants have been identified, and the functional analysis of senescence has been achieved [1]. Moreover, major aging and leaf color genes have been cloned successfully, providing genetic resources for rice molecular-assisted breeding [1].

Genetic changes in plant cells are often the primary cause of leaf color mutations, although the underlying molecular mechanisms are complex and poorly understood. Many endogenous and environmental factors, such as hormone levels, sunshine (light), temperature, drought, mineral nutrition, and cultivation procedures, can affect plant leaf color [1,7]. In addition, leaf color mutations can occur through natural and artificially induced mutagenesis [1,2]. Currently, the most used approaches for identifying genes underlying leaf color changes are RNA-seq and map-based cloning [8,9]. The discovery, cloning, and functional characterization of these mutant genes are crucial steps toward understanding the molecular processes governing changes in leaf color, photosynthesis, pigment synthesis, senescence, photomorphogenesis, and stress response.

*D. officinale* (also called *D. catenatum*) is an Orchid with high medicinal and ornamental values primarily distributed in the tropics and subtropics in South Asia and Oceania [10,11]. Orchids represent about 10% of all seed plant species, have great economic value, and are of specific scientific interest because of their renowned flowers and ecological adaptations [11]. *Dendrobium* is the third largest genus of Orchidaceae and contains approximately 1450 species, characterized by a fleshy stem with abundant polysaccharides and growing in diverse habitats [11]. *D. officinale* is a perennial herb valuable in Chinese traditional medicine. In Chinese pharmacopeia, the herb is reported to nourish the stomach, promote hydration, nourish yin and antipyretic, and treat diabetes, cardiovascular diseases, and gastrointestinal diseases [12,13]. In addition, it produces fresh and elegant flowers, various flower colors, and beautiful postures that have gained a high ornamental value worldwide [13]. Due to its higher medicinal and ornamental potentialities, *D. officinale* is frequently adulterated with other *Dendrobium* species in the market. Its economic value has received considerable attention [13]. Therefore, understanding leaf color mutation mechanisms will contribute to developing diverse attractive *D. offficinale* genotypes and deepen our knowledge of plant physiology and metabolism. In previous studies, we combined physiological and comparative transcriptomics analysis of LCMs of *D. officinale* and unveiled that variation in leaf colors is associated with a significantly reduction in the number of chloroplasts and a decrease of chlorophyll and carotenoid contents [14]. Furthermore, we found that the photosynthetic efficiency of LCMs is greatly influenced by light intensity [15]. Importantly, we have identified key DEGs (differentially regulated genes) related to variation in leaf color [14]. Unfortunately, we could not validate these DEGs as potential candidate genes and perform functional investigations due to the unavailability of a reliable RG for qRT-PCR normalization.

Currently, qRT-PCR analysis is the widely used method to verify the reliability of transcriptome data due to its high sensitivity, accuracy, specificity, and reproducibility [16,17,18,19,20]. It is also applied to quantify the expression of genes and to gain insight into the regulation of metabolic pathways. However, its accuracy relies on various factors, including the integrity of initial samples, quality of RNA, primers specificity, reliability of RGs, and the efficiency of the reverse transcription and amplification [21]. Of them, the suitability of RGs is very critical. The selection of inappropriate RG(s) will cause erroneous results by qRT-PCR analysis, resulting in wrong conclusions [22]. Therefore, using one or more stable RGs as the calibration standard(s) is recommended [20,23]. In many plants, reliable RGs are often selected from housekeeping genes with stable expressions, such as actin (*ACT*), polyubiquitin (*UBQ*), glyceraldehyde 3-phosphate dehydrogenase (*GAPDH*), elongation factor (*EF*), 18S ribosomal RNA (*18S rRNA*), Tubulin (*TUB*), etc. [23,24,25,26]. For instance, *actin2* and *18S rRNA* are used to calibrate the expression levels of leaf color-related genes in *Lilium regale* and wheat, respectively. Notably, a reliable RG should display stable expressions at different developmental stages or under diverse experimental conditions in all plant organs [18,27,28]. But, other studies in many species have revealed that RGs do not always exhibit stable expression levels, specifically under changing environments [28,29,30]. Therefore, it is essential to identify stable and reliable RG(s) for specific traits and experimental conditions in each plant species [18,31,32].

In this study, based on the previously released RNA-seq data [14], we selected ten candidate RGs and investigated their expression stability using common statistical algorithms (BestKeeper, GeNorm, and NormFinder). As a result, we identified and validated the most suitable RG for qRT-PCR normalization of leaf color-related genes in *D. officinale*. Our findings provide optimal RG for analyzing the expression of leaf color genes in *D. officinale*. Moreover, they will promote genomics studies on LCMs in *D. officinale* and contribute to understanding color mutation in plants.

## 2. Materials and Methods

### 2.1. Plant Materials and Sampling

**Figure 1:**
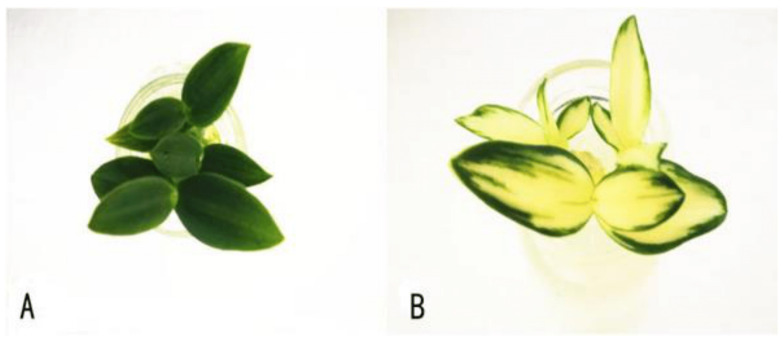
Phenotypic characteristics of *D. officinale* (**A**) and its yellow leaf mutant (**B**).

The wild-type (green leaf, CK) and a leaf color-mutant (yellow) *D. officinale* tissue culture materials were used in this study (Figure 1). When the tissue cultures were at the seedlings stage, leaves were sampled from ten plants of each genotype, mixed, and immediately frozen in liquid nitrogen. Subsequently, the collected samples were stored at −80 °C in a refrigerator until further experimentations. Three biological and experimental repeats were achieved for each analysis.

### 2.2. Selection of Candidate Internal RGs and Chlorophyll Pathway-Related Genes

**Figure 2:**
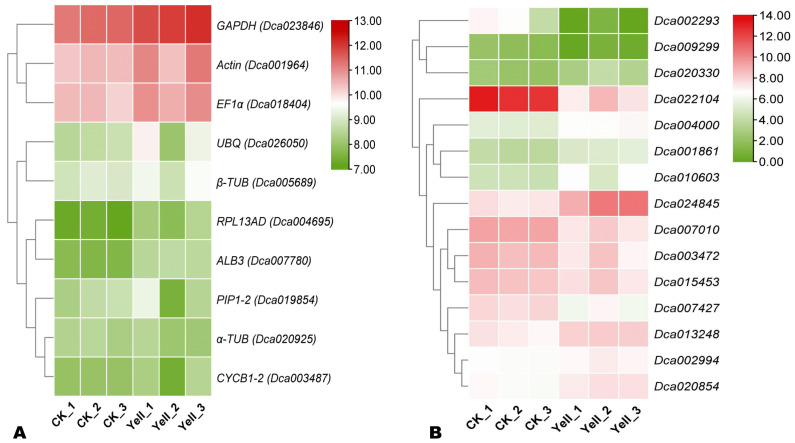
Expression patterns of candidate RGs (**A**) and selected chlorophyll pathway-related genes (**B**) based on Log2FPKM.

We previously analyzed the transcriptome of wild-type and leaf color-mutant of *D. officinale* [14]. Based on the relative stability of the expression patterns of genes (FPKM values) in all groups, ten commonly used internal RGs, including *Actin* (Actin 7), *UBQ* (polyubiquitin), *GAPDH* (3-Glyceraldehyde phosphate dehydrogenase), *EF1α* (elongation factor α), *β-TUB* (β-Tubulin), *α-TUB*, *RPL13AD* (60S ribosomal protein l13a-4), *PIP1-2* (water protein channel pip1-2), *ALB3* (intimal protein ppf-1), and *CYCB1-2* (Cyclin B1-2) were screened out as potential RGs for analyzing the relative expression of leaf color-related genes. The expression patterns and FPKM values of these genes are shown in Figure 2A and Table S1, respectively. In addition, based on the functional annotation of DEGs (differentially expressed genes) from the transcriptomics analysis [14], we selected fifteen genes assigned to pigment synthesis (chlorophyll synthesis pathway) for validation of the most reliable RG (Figure 2B, Table S2). Eight of these genes (*Dca001861*, *Dca002994*, *Dca004000*, *Dca010603*, *Dca013248*, *Dca020330*, *Dca020854*, and *Dca024845*) were up-regulated in yellow (Figure 2B, Table S2). While the remaining seven (*Dca002293*, *Dca003472*, *Dca007010*, *Dca007427*, *Dca009299*, *Dca015453*, and *Dca022104*) were down-regulated (Figure 2B, Table S2). Primer Premier 5.0 software was used to design specific primers for each gene. Primers of the ten potential RG and fifteen genes are presented in Table 1 and Table S3, respectively.

### 2.3. Total RNA Extraction and cDNA Synthesis

Total RNA from all samples was extracted using the RNAprep pure polysaccharide polyphenol plant total RNA extraction kit according to the manufacturer’s instructions. The extracted RNA concentration and quality (OD_260_/OD_280_ value) were assessed with Nanodrop 2000 spectrophotometer (Thermo Scientific, Waltham, MA, USA). Finally, the integrity of total RNA was confirmed through electrophoresis (1% agarose gel).

The GeneAmp^®^ PCR System 9700 (Applied Biosystems, Foster City, CA, USA) was used for cDNA construction. The reverse transcription was achieved using Hiscript II QRT super mix according to the manufacturer’s instructions for users. The reaction conditions were 15 min at 42 °C and 5 s at 85 °C. Each synthesized cDNA was ten-time diluted (with nuclease-free water) and stored in the refrigerator at −20 °C.

### 2.4. qRT-PCR Analysis and Validation of the Most Reliable RG

The qRT-PCR was conducted on Lightcycle^®^ 480 type II fluorescent quantitative PCR (Roche, Indianapolis, USA) using the quantifast^®^ SYBR^®^ green PCR kit and following instructions by the manufacturer. The PCR reaction was a mixture of cDNA 1 μL; Upstream and downstream primers (10 μMol·L^−1^) 0.2 μL for each; 2× QuantiFast^®^ SYBR^®^ Green PCR Master Mix 5 μL; and Nuclease-free H_2_O 3.6 μL. The reaction procedure was as follows: pre-denaturation at 95 °C for 5 min; denaturation at 95 °C for 10 s (40 cycles); and annealing at 60 °C for 30 s. Three technical and biological repetitions were set for each gene. At the end of the cycle, the melting curve was used to detect the product specificity, and each gene’s Ct (cycle threshold) values were computed automatically and saved [24,33].

The reliability of the most reliable RG was confirmed through qRT-PCR analysis of the relative expression of the fifteen selected chlorophyll pathway-related genes, followed by expression level calculation via the 2^−∆∆Ct^ method [34]. The internal control was the most stable RG.

### 2.5. Stability and Statistical Analyses

The Ct values were considered as relative quantities to perform gene expression stability analysis [23,33]. Three commonly used software, including NormFinder [16,18], BestKeeper [34], and geNorm [31]. The software were run following instructions in their respective manuals. Finally, we applied the GM (geometric mean) method to fit the results from the three software and generate a comprehensive stability ranking for the candidate RGs [35]. Data Processing System, GraphPad Prism v9.0.0121 (GraphPad 159 Software Inc., La Jolla, CA, USA), and Microsoft Excel 2016 were used for data analysis and graphing [35,36]. Statistical differences were obtained via an independent *t*-test at *p* < 0.05. Finally, we used TBtools software to construct the heatmap of genes [37].

## 3. Results

### 3.1. Primers Specificity and Expression Profiles Analyses of Candidate RGs

**Figure 3:**
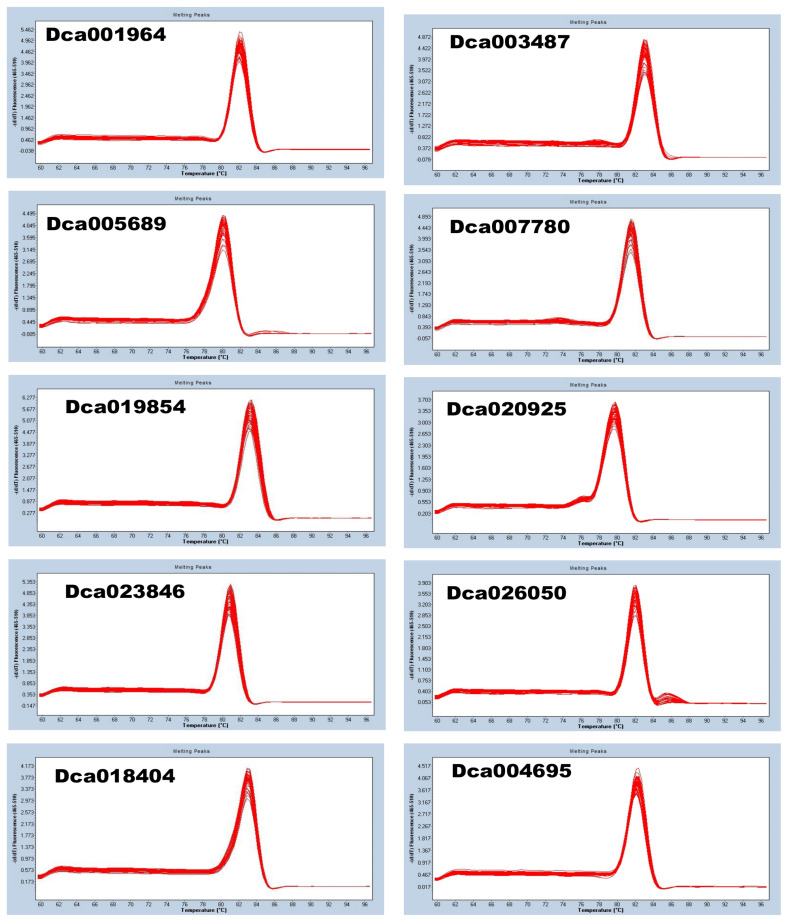
Melting curves of the ten candidate RGs (reference genes).

To uncover a suitable RG for normalizing the relative expression levels of leaf color-related genes in *D. officinale* via qRT-PCR, we selected ten candidate RGs (Table S1) from transcriptome data and subjected them to qRT-PCR analysis. The melting curves showed that the primers of each potential RG were highly specific (Figure 3). All ten genes exhibited a single peak with no primer dimer, and all amplicons showed good repeatability, confirming the accuracy, reliability, and specificity of primers.

**Figure 4:**
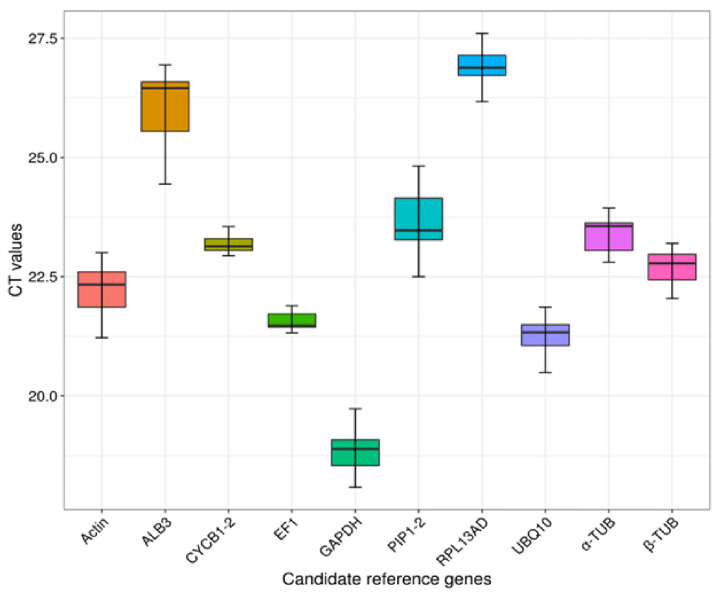
qRT-PCR Ct values for all candidate reference genes in all samples.

In order to assess the expression levels of each potential RGs, we automatically computed their respective Ct values. The results showed that the Ct values of the ten genes ranged from 18.858 (*GAPDH*) to 26.915 (*RPL13AD*) on average (Figure 4). The average Ct value of *actin-7*, *UBQ10*, *EF1α*, *β-TUB*, *α-TUB*, *PIP1-2*, *ALB3*, and *CYCB1-2* was 22.241, 21.288, 21.493, 22.678, 23.386, 23.571, 26.069, and 23.230, respectively. *CYCB1-2* exhibited the least expression variation from 22.94 to 23.93, followed by *EF1α* from 20.84 to 22.20 (Figure 4). The summary of the variation in expression levels (Ct values) of all candidate RGs is presented in Table S4. The coefficient of variation (CV) of *Actin*, *UBQ10*, *GAPDH*, *EF1α*, *β-TUB*, *α-TUB*, *RPL13AD*, *PIP1-2*, *ALB3*, *CYCB1-2* was 2.33%, 2.13%, 2.18%, 1.57%, 1.54%, 1.45%, 1.30%, 2.82%, 3.11%, 1.14%, respectively (Table S4).

### 3.2. Stability Analysis of Candidate Internal RGs

**Figure 5:**
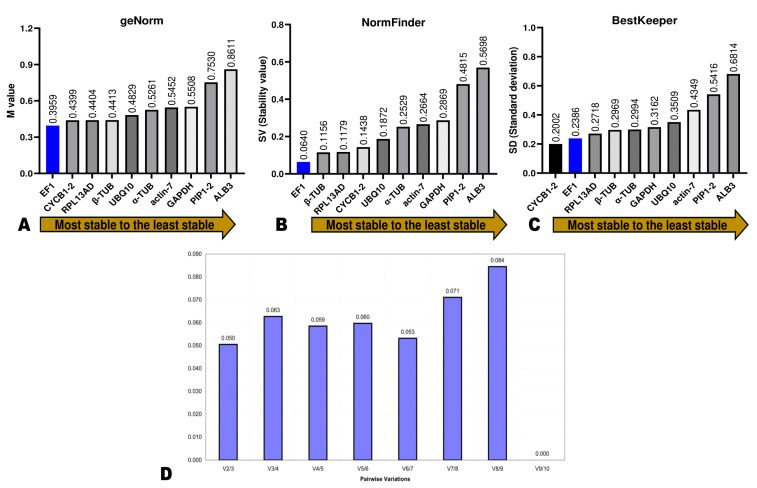
Expression stability of candidate RGs (reference genes) by geNorm analysis (**A**), NormFinder (**B**), and BestKeeper (**C**). (**D**) The pairwise variation values (Vn/Vn + 1, V) of candidate RGs.

To determine the stability rankings of the ten potential RGs, three independent software (BestKeeper, GeNorm, and NormFinde) were used, and the results were integrated via the GM method to obtain the comprehensive stability ranking. Firstly, we used geNorm to compute the expression stability measured (M) of each potential RG based on average pairwise expression ratios. A gene can be selected as an internal RG if its M value is below 1.5, and the lower the M value, the more the gene is stable [24]. The analysis by geNorm showed that the M values of all ten genes were >0.9, indicating they both met the basic requirements for an internal RG (Figure 5A). *EF1α* was the most stable gene, with an M value of 0.395, followed by *CYCB1-2* (M value of 0.439) (Figure 5A). *PIP1-2* and *ALB3* recorded the highest stability M values of 0.753 and 0.861, respectively. Usually, a single RG is insufficient to achieve high accuracy. Accordingly, sometimes using two or more RGs for precise and reliable normalization is recommended. The optimal number of RGs is determined by pairwise variation value (Vn/n + 1, V value). If Vn/n + 1 ≥ 0.15, the adequate number of RGs equals n + 1. In contrast, if Vn/n + 1 < 0.15, the number is n [23,31]. The V value of all pairwise comparisons was less than 0.15 (Figure 5D), indicating two RGs might be required. Thus, the M values indicate *EF-1α* + *CYCB1-2* is the best combination for accurate normalization of leaf color-related genes’ expression levels in *D. officinale*.

NormFinder serves to compute the SV (stability value) of RGs and unveil the optimal number of RGs for precise normalization through analysis of intra- and inter-group variations [24]. The most stable gene is one that exhibits a lower expression level than the average SVs. The stability ranking by NormFinder and GeNorm analyses were somewhat similar, with a little variation in the classification of the top four genes (Figure 5B). Interestingly, *EF1α* (SV = 0.064) occupied the first rank, confirming it was the most stable RG. β-*TUB* (0.115), *RPL13AD* (0.117), and *CYCB1-2* (0.143) ranked second, third, and fourth in terms of stability by NormFinder, respectively (Figure 5B). *PIP1-2* (SV = 0.481) and *ALB3* (SV = 0.57) occupy the last ranking positions as per the GeNorm results.

BestKeeper helps to evaluate the stability index of RGs by calculating mainly the SD (standard deviation) [38]. A gene is stable if its SD is lower than 1.0 [24,38]. The results by BestKeeper indicated that the SD of all ten genes was lower than 0.7, supporting that they are suitable for use as internal RGs (Figure 5C). *CYCB1-2* (SV = 0.2002) and *EF1α* (SV = 0.2386) ranked first and second in terms of stability, respectively (Figure 5C). Both three software revealed that *PIP1-2* and *ALB3* were the least stable genes.

We applied the GM method to integrate the results from the three software and determine the comprehensive stability ranking of the ten RGs. As presented in Table 2, GM values confirmed that *EF1α* was the most suitable and reliable RG, followed by *CYCB1-2*, *RPL13AD*, *β-TUB*, *UBQ10*, *α-TUB*, *GAPDH*, *Actin*, *PIP1-2*, and *ALB3*.

### 3.3. Validation of EF1α as the Most Reliable and Stable Internal RG

**Figure 6:**
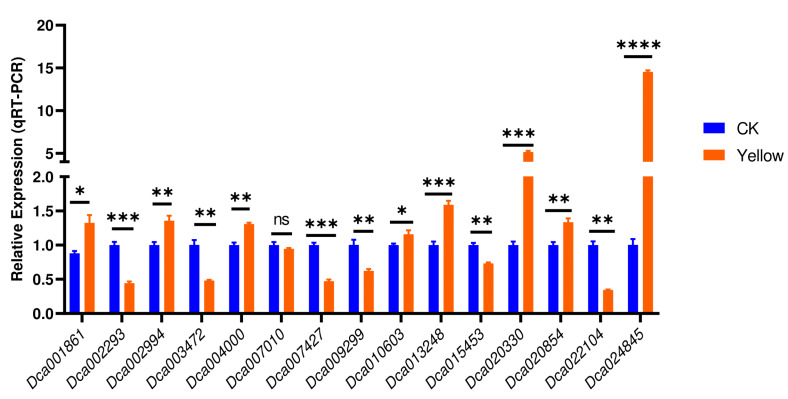
The relative expression levels of fifteen chlorophyll pathway-related genes via normalization with the most suitable RG (reference gene) *EF1α*. *, **, ***, **** indicate statistical differences at *p* < 0.05, 0.01, 0.001, and 0.0001, respectively.

To confirm the reliability and stability of *EF1α* for accurate normalization of relative expression levels of leaf color-related genes, we investigated the expression of fifteen selected genes from the chlorophyll biosynthesis pathway (Figure 2B, Table S2) via qRT-PCR. As shown in Figure 6, the expression patterns of the targeted genes via qRT-PCR and RNA-Seq were consistent with identical regulation patterns. *Dca001861*, *Dca002994*, *Dca004000*, *Dca010603*, *Dca013248*, *Dca020330*, *Dca020854*, and *Dca024845* were significantly up-regulated as per the results of transcriptomics analysis (Figure 6). Among them, *Dca024845* and *Dca020330* were the most significantly up-regulated, suggesting they might play crucial roles in yellow leaf development in *D. officinale*.

## 4. Discussion

Leaf color is a critical agronomic trait, and its variation significantly affects global plant metabolism. Principally, leaf color mutations induce less photosynthetic efficiency, causing poor growth, reduced yield, and important economic losses [1,2]. Accordingly, leaf color mutants have become materials of considerable research interest. They are ideal for studying variations in plant metabolism and physiology, especially the molecular mechanisms governing chloroplast biogenesis and differentiation, pigment synthesis and accumulation, photosynthesis, diverse stress response, etc. [1,2]. In *D. officinale*, previous studies have shown that leaf color mutation is associated with a reduction in plant height, stem diameter, and the number of chloroplasts; low chlorophyll content; and altered chloroplast structure [14,15]. In addition, they have provided insight into the underlying molecular mechanisms through comparative transcriptome analysis and unveiled DEGs [14]. However, the identified potential candidate genes have not been validated, and functional characterizations are yet to be conducted due to the unavailability of a reliable RG for qRT-PCR analysis. Thus, the present study took advantage of the RNA-seq data to comprehensively select candidate RGs, examine their stability, and validate the most stable to promote genomics studies on leaf color mutants in *D. officinale*. This approach has been widely used in several plants to identify suitable RGs for specific traits and experimental conditions [20,24,39,40,41].

The efficiency of qRT PCR results depends on the reliability of internal RGs. Only a stable expression of internal RG can guarantee the accuracy of the results. Previous studies have demonstrated that internal RGs are species-specific, and their stability varies according to the traits and experimental conditions [20,24,39,40,41]. For instance, different RGs have been identified for studying traits related to pigment synthesis in different plant species. In *Catalpa fargesii*, *CfMADH* and *CfEF-1* have been identified as the most reliable RGs to be used individually or in combination for normalizing the expression levels of leaf color-related genes [40]. In *Lagerstroemia indica*, *LiEF1α-2* and *LiEF1α-1* were identified as the most suitable for analyzing the expression levels of leaf color-related genes [42]. In wheat, 18S rRNA is used as the internal RG to calibrate the expression of leaf color-related candidate genes [26]. In *Lilium regale*, *LrActin2* was identified as the best RG for normalizing photosynthesis-related candidate genes’ expression levels [25]. Herein, we investigated ten genes, including *EF1α*, *CYCB1-2*, *RPL13AD*, *β-TUB*, *UBQ10*, *α-TUB*, *GAPDH*, *Actin*, *PIP1-2*, and *ALB3.* The melting curves showed that all ten genes exhibited a single peak with no primer dimer, and all amplicons showed good repeatability. These results indicate that the designed primers were highly specific, accurate, and reliable. These primers could be directly employed in future studies. The coefficient of variation (CV) of the Ct values of all ten genes was lower than 3%, confirming the repeatability of the experiment. Expression stability analysis via GeNorm, NormFinder, and BestKeeper revealed that these ten genes could be used as internal RGs to calibrate leaf color-related genes’ expression levels in *D. officinale*. The comprehensive ranking analysis showed that *EF1α* was the most stable.These results suggest that *EF1α* is the most reliable RG for calibrating the expression levels of leaf color-related genes in *D. officinale*. Using this RG may promote the molecular dissection of the regulatory network of leaf color mutations in *D. officinale*. We did not investigate the expression stability of these RGs in other plant organs since this study aimed to identify a reliable RG for leaf color specifically.

To verify the suitability and reliability of *EF1α*, we analyzed fifteen chlorophyll pathway-related DEGs’ expression levels via qRT qPCR. The expression patterns of the targeted genes via *EF1α* normalization were consistent with the transcriptome sequencing results. This result further confirms the accuracy of using *EF1α* as the primary RG for qRT-PCR calibration of leaf color-related genes’ expression levels in *D. officinale.* Furthermore, of the fifteen analyzed genes, *Dca024845* and *Dca020330* were the most significantly up-regulated in the yellow leaf mutant genotype. Accordingly, these genes may represent key candidate genes for deciphering chlorophyll synthesis-related molecular mechanisms in *D. offinale*. In rice, most leaf color mutants are directly or indirectly involved in chlorophyll metabolism [1]. Any defects in the development of chloroplasts also impair the stability of chlorophyll and other photosynthetic pigments, eventually changing the green color of the leaf [1]. In general, leaf color mutation is caused by complex mechanisms, including abnormal chlorophyll metabolism pathways (mutations of genes related to chlorophyll biosynthesis or degradation pathways and mutations of genes related to heme metabolism pathway), abnormal chloroplast development and differentiation, defects in carotenoid metabolism, abnormal anthocyanin biosynthesis and degradation (mutations of structural and regulation genes) [2]. Therefore, functional characterization of these genes is required to reveal their roles and gain insight into leaf coloring molecular mechanisms in *D. officinale*. With the rapid development of genomics tools, the discovery of this RG may accelerate the molecular dissection of leaf coloring mechanisms in *D. officinale*.

## 5. Conclusions

In summary, this study investigates ten RGs for qRT-PCR normalization of expression levels of leaf color-related candidate genes in *D. officinale.* Stability rankings analysis showed that all ten genes, including Actin (*Actin*), polyubiquitin (*UBQ*), glyceraldehyde-3-phosphate dehydrogenase (*GAPDH*), elongation factor 1-α (*EF1α*), β-tubulin (*β-TUB*), α-tubulin (*α-TUB*), 60S ribosomal protein L13-1 (*RPL13AD*), aquaporin PIP1-2 (*PIP1-2*), Intima protein (*ALB3*) and Cyclin (*CYCB1-2*) met the requirement of an RG. Of them, *EF1α* was the most stable, and its accurability was confirmed through the qRT-PCR calibration of the expression levels of fifteen chlorophyll pathway-related DEGs. The regulation patterns of the fifteen chlorophyll pathway-related genes were identical to the RNA-seq results. *Dca024845* and *Dca020330* were the most significantly up-regulated genes in the yellow leaf mutant genotype. Therefore, they were proposed as candidate genes for dissecting leaf coloring molecular mechanisms in *D. officinale*. Our results provide key genetic resources for validating leaf color mutants-related candidate genes and perform functional genomics studies. Furthermore, they may provide comprehensive guidelines for uncovering suitable RGs in other plant species.

## Figures and Tables

**Table 1:** Primers of the candidate reference genes for qRT-PCR analysis.

No	Genes	Primers (5′-3′)	Product Length (bp)	Tm Value/°C
1	*Actin*	F: CCCTTTATGCTAGTGGTCGAA; R: CCTGAGAATCGCATGTGGTA	110	60
2	*UBQ*	F: GCCGACTACAACATCCAGAA; R: TGATGGTCTTGCCTGTGAG	100	60
3	*GAPDH*	F: ATTTCTTGGGTGACAGCAG; R: TGTCATACCAAGCCACGA	93	60
4	*EF1α*	F: GCTGTGAAGGATCTAAAGCG; R: TGGGAGGTAAAGTTGGCG	83	60
5	*β-TUB*	F: GGCAAGATGAGCACCAAAG; R: GGAATCCACTCCACGAAG	83	60
6	*α-TUB*	F: GAGAGGTTGTCCGTGGACTA; R: CTACTGCTGTGGAAACCTG	82	60
7	*RPL13AD*	F: CGGGCAAAGGTTGCATAC; R: CGAGTTTCTCTTCAGCTGTT	82	60
8	*PIP1-2*	F: TTGGCGCTGAGATCATCG; R: TGGAACATGAGAGTCCCTG	92	60
9	*ALB3*	F: GTTGCTAGGGTTCGGATGA; R: AAGTAATTCCGCCCAAGTC	82	60
10	*CYCB1-2*	F: TACCAAGATGCCCTTCGC; R: GTCTCCCGCAGATATTCCAG	83	60

**Table 2:** The comprehensive ranking of candidate RGs (reference genes) stability by GM method.

Ranking	Reference Genes	Geomean of Ranking Values
1	*EF1α*	1.260
2	*CYCB1-2*	2.000
3	*RPL13AD*	3.000
4	*β-TUB*	3.175
5	*UBQ10*	5.593
6	*α-TUB*	5.646
7	*GAPDH*	7.268
8	*Actin*	7.319
9	*PIP1-2*	9.000
10	*ALB3*	10.000

## Data Availability

All datasets analyzed or generated during the study are included in this manuscript and its supplementary file.

## Supplementary Materials

The following supporting information can be downloaded at: https://www.mdpi.com/article/10.3390/genes14051112/s1, Table S1: List of the ten candidate reference genes with their FPKM expression values; Table S2: List of the fifteen chlorophyll pathway-related genes with their FPKM expression values; Table S3: Primers of the fifteen chlorophyll pathway-related genes for qRT-PCR analysis; Table S4: Summary of the qRT-PCR Ct value of ten candidate genes.

## References

[1] Li W., Zhang Y., Mazumder M.A.R., Pan R., Akhter D. (2022). Research progress on rice leaf color mutants. Crop Des..

[2] Zhao M.H., Li X., Zhang X.X., Zhang H., Zhao X.Y. (2020). Mutation mechanism of leaf color in plants: A review. Forests.

[3] Jung K.H., Hur J., Ryu C.H., Choi Y., Chung Y.Y., Miyao A., Hirochika H., An G. (2003). Characterization of a rice chlorophyll-deficient mutant using the T-DNA gene-trap system. Plant Cell Physiol..

[4] Sheng P., Tan J., Jin M., Wu F., Zhou K., Ma W., Heng Y., Wang J., Guo X., Zhang X. (2014). Albino midrib 1, encoding a putative potassium efflux antiporter, affects chloroplast development and drought tolerance in rice. Plant Cell Rep..

[5] Tanaka A., Tanaka R. (2006). Chlorophyll metabolism. Curr. Opin. Plant Biol..

[6] Falbel T.G., Staehelin L.A. (1996). Partial blocks in the early steps of the chlorophyll synthesis pathway: A common feature of chlorophyll b-deficient mutants. Physiol. Plant..

[7] Brestic M., Zivcak M., Kunderlikova K., Allakhverdiev S.I. (2016). High temperature specifically affects the photoprotective responses of chlorophyll b-deficient wheat mutant lines. Photosynth. Res..

[8] Lee S., Masclaux-Daubresse C. (2021). Current understanding of leaf senescence in rice. Int. J. Mol. Sci..

[9] Fu M., Cheng S., Xu F., Chen Z., Liu Z., Zhang W., Zheng J., Wang L. (2021). Advance In Mechanism Of Plant Leaf Colour Mutation. Not. Bot. Horti Agrobot. Cluj-Napoca.

[10] Qi L., Shi Y., Li C., Liu J., Chong S.-L., Lim K.-J., Si J., Han Z., Chen D. (2022). Glucomannan in *Dendrobium catenatum*: Bioactivities, Biosynthesis and Perspective. Genes.

[11] Zhang G.-Q., Xu Q., Bian C., Tsai W.-C., Yeh C.-M., Liu K.-W., Yoshida K., Zhang L.-S., Chang S.-B., Chen F. (2016). The *Dendrobium catenatum* Lindl. genome sequence provides insights into polysaccharide synthase, floral development and adaptive evolution. Sci. Rep..

[12] Xi H., Liu J., Li Q., Chen X., Liu C., Zhao Y., Yao J., Chen D., Si J., Liu C. (2021). Genome-wide identification of Cellulose-like synthase D gene family in *Dendrobium catenatum*. Biotechnol. Biotechnol. Equip..

[13] Yuan Y., Zuo J., Zhang H., Zu M., Yu M., Liu S. (2022). Transcriptome and metabolome profiling unveil the accumulation of flavonoids in *Dendrobium officinale*. Genomics.

[14] Cao H., Li H., Miao Z., Fu G., Yang C., Wu L., Zhao P., Shan Q., Ruan J., Wang G. (2017). The preliminary study of leaf-color mutant in *Dendrobium officinale*. J. Nucl. Agric. Sci..

[15] Ji Y., Yang W., Li H., Cao H., Lu L., Tian M., Sun D., Li D. (2020). Study on chloroplast ultrastructure, photosynthetic pigments, and chlorophyll fluorescence characteristics of leaf color mutants in *Dendrobium officinale* Kimura et Migo. Plant Sci. J..

[16] Cao A., Shao D., Cui B., Tong X., Zheng Y., Sun J., Li H. (2019). Screening the reference genes for quantitative gene expression by RT-qPCR during SE initial dedifferentiation in four *Gossypium hirsutum* cultivars that have different SE capability. Genes.

[17] Huggett J., Dheda K., Bustin S., Zumla A. (2005). Real-time RT-PCR normalisation; strategies and considerations. Genes Immun..

[18] Liu Q., Qi X., Yan H., Huang L., Nie G., Zhang X. (2018). Reference gene selection for quantitative real-time reverse-transcriptase PCR in annual ryegrass (*Lolium multiflorum*) subjected to various abiotic stresses. Molecules.

[19] Wang L., Dossou S.S.K., Wei X., Zhang Y., Li D., Yu J., Zhang X. (2020). Transcriptome dynamics during black and white sesame (*Sesamum indicum* L.) seed development and identification of candidate genes associated with black pigmentation. Genes.

[20] Wu Y., Zhou J., Liu Y., Gu Y., Zhang H., Ahmad F., Wang G., Ren L. (2022). Selection and Validation of Reliable Reference Genes for qRT-PCR Normalization of *Bursaphelenchus xylophilus* from Different Temperature Conditions and Developmental Stages. Appl. Sci..

[21] Kou X., Zhang L., Yang S., Li G., Ye J. (2017). Selection and validation of reference genes for quantitative RT-PCR analysis in peach fruit under different experimental conditions. Sci. Hortic..

[22] Dheda K., Huggett J.F., Chang J.S., Kim L.U., Bustin S.A., Johnson M.A., Rook G.A.W., Zumla A. (2005). The implications of using an inappropriate reference gene for real-time reverse transcription PCR data normalization. Anal. Biochem..

[23] Xu L., Xu H., Cao Y., Yang P., Feng Y., Tang Y., Yuan S., Ming J. (2017). Validation of reference genes for quantitative real-time pcr during bicolor tepal development in Asiatic hybrid lilies *(Lilium* spp.). Front. Plant Sci..

[24] Chen C., Wu J., Hua Q., Tel-Zur N., Xie F., Zhang Z., Chen J., Zhang R., Hu G., Zhao J. (2019). Identification of reliable reference genes for quantitative real-time PCR normalization in pitaya. Plant Methods.

[25] Du W., Hu F., Yuan S., Liu C. (2019). Selection of reference genes for quantitative real-time PCR analysis of photosynthesis-related genes expression in *Lilium regale*. Physiol. Mol. Biol. Plants.

[26] Wu H., Shi N., An X., Liu C., Fu H., Cao L., Feng Y., Sun D., Zhang L. (2018). Candidate Genes for Yellow Leaf Color in Common Wheat (*Triticum aestivum* L.) and Major Related Metabolic Pathways according to Transcriptome Profiling. Int. J. Mol. Sci..

[27] Wan H., Zhao Z., Qian C., Sui Y., Malik A.A., Chen J. (2010). Selection of appropriate reference genes for gene expression studies by quantitative real-time polymerase chain reaction in cucumber. Anal. Biochem..

[28] Liang L., He Z., Yu H., Wang E., Zhang X., Zhang B., Zhang C., Liang Z. (2020). Selection and Validation of Reference Genes for Gene Expression Studies in *Codonopsis pilosula* Based on Transcriptome Sequence Data. Sci. Rep..

[29] Li J., Huang H., Shan T., Pang S. (2019). Selection of reference genes for real-time RT-PCR normalization in brown alga *Undaria pinnatifida*. J. Appl. Phycol..

[30] Yi S., Qian Y., Han L., Sun Z., Fan C., Liu J., Ju G. (2012). Selection of reliable reference genes for gene expression studies in *Rhododendron micranthum* Turcz. Sci. Hortic..

[31] Derveaux S., Vandesompele J., Hellemans J. (2010). How to do successful gene expression analysis using real-time PCR. Methods.

[32] Ren R., Dai P.-H., Li M., Liu Z., Cao F. (2016). Selection and stability evaluation of reference genes for real-time quantitative PCR in dove tree *(Davidia involucrata*). Plant Physiol. Commun..

[33] Ma L., Cui G., Wang X., Jia W., Duan Q., Du W., Wang J. (2017). Cloning and expression analysis of catalase (Ls-Cat1) gene in *Lilium sargentiae* Wilson. J. Nucl. Agric. Sci..

[34] Livak K.J., Schmittgen T.D. (2001). Analysis of Relative Gene Expression Data Using Real-Time Quantitative PCR and the 2^−ΔΔCT^ Method. Methods.

[35] Li F., Cheng Y., Ma L., Li S., Wang J. (2022). Identification of reference genes provides functional insights into meiotic recombination suppressors in *Gerbera hybrida*. Hortic. Plant J..

[36] Tang Q.Y., Zhang C.X. (2013). Data Processing System (DPS) software with experimental design, statistical analysis and data mining developed for use in entomological research. Insect Sci..

[37] Chen C., Chen H., Zhang Y., Thomas H.R., Frank M.H., He Y., Xia R. (2020). TBtools: An Integrative Toolkit Developed for Interactive Analyses of Big Biological Data. Mol. Plant.

[38] Pfaffl M.W., Tichopad A., Prgomet C., Neuvians T.P. (2004). Determination of stable housekeeping genes, differentially regulated target genes and sample integrity: BestKeeper—Excel-based tool using pair-wise correlations. Biotechnol. Lett..

[39] Ma K.S., Li F., Liang P.Z., Chen X.W., Liu Y., Gao X.W. (2016). Identification and validation of reference genes for the normalization of gene expression data in qRT-PCR Analysis in *Aphis gossypii* (Hemiptera: Aphididae). J. Insect Sci..

[40] Yang Y.Y., Zhao L.J., Yang G.J., Zhang Y., Fu P.Y., Hu J.W., Liu Y., Wang N. (2022). Selection and Validation of Reference Genes for Leaf Color Phenotype in “Maiyuanjinqiu”, a *Catalpa fargesii* Variety, by qRT-PCR. For. Res..

[41] Zhang J.R., Feng Y.Y., Yang M.J., Xiao Y., Liu Y.S., Yuan Y., Li Z., Zhang Y., Zhuo M., Zhang J. (2022). Systematic screening and validation of reliable reference genes for qRT-PCR analysis in Okra (*Abelmoschus esculentus* L.). Sci. Rep..

[42] Chen M., Wang Q., Li Y., Gao L., Lv F., Yang R., Wang P. (2021). Candidate reference genes for quantitative gene expression analysis in *Lagerstroemia indica*. Mol. Biol. Rep..

